# High expression of *ZNF703* independent of amplification indicates worse prognosis in patients with luminal B breast cancer

**DOI:** 10.1002/cam4.88

**Published:** 2013-05-22

**Authors:** Inga Reynisdottir, Adalgeir Arason, Berglind O Einarsdottir, Haukur Gunnarsson, Johan Staaf, Johan Vallon-Christersson, Goran Jonsson, Markus Ringnér, Bjarni A Agnarsson, Kristrun Olafsdottir, Rainer Fagerholm, Thorbjorg Einarsdottir, Gudrun Johannesdottir, Oskar Thor Johannsson, Heli Nevanlinna, Ake Borg, Rosa Bjork Barkardottir

**Affiliations:** 1 Department of Pathology, Landspitali-University Hospital101, Reykjavik, Iceland; 2 BMCFaculty of Medicine, University of Iceland101, Reykjavik, Iceland; 3 Department of OncologyClinical Sciences, Lund UniversityBarngatan 2B, SE 22185, Lund, Sweden; 4 CREATE Health Strategic Center for Translational Cancer Research, Lund UniversityBMC C13, SE 22184, Lund, Sweden; 5 Department of Oncology, Landspitali-University Hospital101, Reykjavik, Iceland; 6 Department of Obstetrics and Gynaecology, University of Helsinki and Helsinki University Central HospitalHelsinki, Finland

**Keywords:** 8p12-p11 amplification, ER positive, luminal tumors, prognosis, tumor progression, ZNF703

## Abstract

Amplification of 8p12-p11 is relatively common in breast cancer and several genes within the region have been suggested to affect breast tumor progression. The aim of the study was to map the amplified 8p12-p11 region in a large set of breast tumors in an effort to identify the genetic driver and to explore its impact on tumor progression and prognosis. Copy number alterations (CNAs) were mapped in 359 tumors, and gene expression data from 577 tumors (359 tumors included) were correlated with CNA, clinical–pathological factors, and protein expression (39 tumors). 8p12-p11 was amplified in 11.4% of tumors. The smallest region of amplification harbored one full-length gene, *ZNF703*. ZNF703 mRNA expression was significantly higher in estrogen receptor (ER)-positive than ER-negative tumors (*P* = 2 × 10^−16^), a reflection of high expression in luminal tumors. Forty-eight percent of tumors with *ZNF703* amplification were luminal B tumors in which the best correlation between DNA copy number and mRNA was seen (*P* = 1.2 × 10^−7^) as well as correlation between mRNA and protein expression (*P* = 0.02). High ZNF703 mRNA correlated with poor survival in patients with ER-positive luminal B tumors (log rank *P* = 0.04). Furthermore, high ZNF703 mRNA expression correlated with poor outcome in patients with *ZNF703* copy number neutral, ER-positive, luminal B tumors (log rank *P* = 0.004). The results support *ZNF703* as the driver gene of the 8p12 amplification and suggest that independent of amplification, high expression of the gene affects prognosis in luminal B tumors.

Our mapping of 8p12-p11 and analyses of ZNF703 mRNA and protein expression in breast tumors support *ZNF703* as an oncogene in luminal B tumors. High *ZNF703* expression, independent of the amplification, correlated with worse prognosis for the breast cancer patients with ER-positive luminal tumors, particularly of the luminal B subtype.

## Introduction

Amplifications are common in breast cancer. Some of the genes within the amplicons are oncogenes that drive the amplification while others are passengers. Amplifications at 8p12-p11 occur in 10–15% of breast tumors 1–6 and have been associated with poor prognosis and shorter survival 3–9. The complex pattern of the 8p12-p11 amplification undoubtedly has delayed the identification of a genetic driver. Four amplicons, A1–A4, have been mapped consistently in the 8p12-p11 region where the telomeric amplicon A1 is most often amplified 3–13. Five genes – *ZNF703*,* ERLIN2*,* PROSC*,* BRF2*, and *RAB11FIP1* – residing within A1 were indicated as potential drivers of the amplicon based on positive correlation between DNA amplification and gene expression 3–13. *ERLIN2* 14 was shown to have transforming properties in breast cancer cell lines as well as *RAB11FIP1* 15, which also had tumorigenic properties in mouse xenograft models. Two studies, in which a large number of breast tumors were used, demonstrated that *ZNF703* meets the criteria as the genetic driver of the A1 amplicon, emerging as a gene with an oncogenic role in luminal B type breast cancer 16–17. This was recently confirmed in a large study of the genome and transcriptome in 2000 breast tumors 18. Alteration of *ZNF703* expression has been shown to affect cellular gene expression allowing cells to evade replication constraints and steer them toward self-renewal rather than differentiation 16–17. Its role in breast cancer progression, or metastasis, was demonstrated in a mouse model of breast cancer where enhanced *ZNF703* expression repressed E-cadherin expression and increased lung metastases 19. Loss of E-cadherin, which promotes cellular adhesion in normal breast epithelial cells, increases the proliferative and invasive potential of breast cancer cells, which can promote epithelial mesenchymal transition and metastasis 20. These functions suggest that *ZNF703* plays an important role in breast cancer formation and progression.

The luminal B subtype of breast tumors along with the luminal A subtype are two of five molecular subtypes that have been defined by gene expression analysis 21–22. The different classes of molecular subtypes predict patient prognosis 22,23 and aid in prediction of response to neoadjuvant treatment in node-negative patients 25. The luminal subtypes constitute the majority of tumors that express the estrogen receptor (ER), which is expressed in two thirds of all breast tumors. As patients with luminal B tumors have a higher risk of relapse than patients with tumors of the luminal A subtype 22,23, there has been an effort to distinguish between them. Tumors of the luminal B subtype are commonly of higher grade compared with luminal A tumors 26, and proliferative genes such as Ki67 27 are more highly expressed 22–24 in luminal B tumors. The luminal tumors can also be distinguished based on their genomic subtypes, where luminal A tumors have fewer copy number alterations (CNAs) – amplifications and losses – than tumors of the luminal B subtype 28.

Our mapping of the 8p12-p11 region in 359 Nordic breast tumors resulted in the identification of *ZNF703* as the only full-length gene within the minimal region of amplification. It was found to be expressed mainly in ER-positive tumors, highest and most frequent in the ER-positive luminal B subtype. In addition, high ZNF703 mRNA levels in luminal B tumors resulted in poor prognosis even in the absence of gene amplification.

## MATERIALS AND METHODS

### Patients and breast tumor samples

The breast tumor samples were obtained from the Department of Pathology, Landspitali-University Hospital in Reykjavik, Iceland, the Department of Oncology, Skåne University Hospital, Lund, Sweden, and from the Helsinki University Central Hospital, Finland. As explained in Jonsson et al. 29, approval was obtained from ethical committees for the Icelandic, Swedish, and Finnish tumor specimens in the respective countries: The Icelandic Data Protection Committee (2001/523 and 2002/463), the National Bioethics Committee of Iceland (99/051 and 99/051_FS1), the regional Ethical Committee in Lund (reg. no. LU240-01 and 2009/658), and the Helsinki University Central Hospital Ethical Committee (207/E9/07).

### Gene expression

Array-GEX for ZNF703 was retrieved for 577 samples from the Gene Expression Omnibus (GEO) dataset GSE25307 29. In order to verify the results, a quantitative real-time polymerase chain reaction (qRT-PCR) was performed on all available tumor samples, which was a subset (*n* = 137) of the tumors from the Icelandic patients (*n* = 161). RNA was extracted from fresh frozen tumors using Trizol, purified by RNeasy Midi purification kit (Qiagen, Hilden, Germany), and 2 μg was reverse transcribed with random hexamer primers using the RevertAid Minus First strand cDNA synthesis kit (Thermo Fisher Scientific, Vilnius, Lithuania). One nanogram of cDNA was used as template. Taqman Gene Expression Assay Hs00228155_m1 was used for *ZNF703* and the TATA-binding protein (*TBP*, 4326322E Applied Biosystems, Nærum, Denmark) was a reference gene. Expression level of the target genes relative to the reference gene was quantified as follows: mRNA expression of target gene = 2^−(mean Ct target − mean Ct reference)^. A correlation was observed between the ZNF703 mRNA values obtained by the two methods (*r* = 0.75, *P* = 2 × 10^−16^).

### BAC array-CGH analysis

Bacterial artificial chromosome (BAC) array-comparative genomics hybridization (CGH) data were obtained for 359 tumors from GEO dataset GSE22133 28, inclusive in the set of 577 samples from the GSE25307 dataset. The BAC clones were mapped by the BACPAC consortium to hg17 in 2004. To analyze CNAs within 8p12-p11, breakpoint analysis was performed using circular binary segmentation (CBS) with α = 0.001 31. The minimum number of BACs per segment was ≥4. Gaps between segments were closed by extending the adjacent segments into the center of the gap. The threshold for loss was set to segmented log_2_ ratio less than −0.32, gain was set to segmented log_2_ ratio ≥0.5, and amplification was set to segmented log_2_ ratio ≥0.85. Loss, gain, and amplification were observed in 34, 29, and 41 tumors, respectively. The 41 tumors showing amplification were used to map the smallest region of overlap (SRO) that was most often amplified.

### Clinical parameters and pathology

Clinical, pathological, and molecular characteristics were retrieved from the GSE25307 dataset 29 and can be seen in Table S1. The histopathology of the tumors was categorized according to the WHO histological classification and the histological grade was determined by the modified Bloom–Richardson system. The ER and progesterone (PR) status was determined as being either positive (staining 1+ to 3+) or negative (no staining).

### Tumor protein extraction, Western blotting, and protein quantification

Protein expression was analyzed in 39 Icelandic breast tumors. The power to detect a correlation using 39 tumors, assuming an effect size of 0.5 and α = 0.05, was 0.9. Twelve tumors were of the luminal A subtype, 10 were luminal B, 4 were normal like, and 6 were basal. The subtype was not available (NA) for six tumors and one tumor originated in another organ and was not relevant. Protein was extracted from approximately 100 μg of breast tumor tissue that had been fresh frozen in liquid N_2_; 5 μg of each tumor sample was subjected to SDS-PAGE (sodium dodecyl sulfate polyacrylamide gel electrophoresis) and Western blotting according to standard protocols using 0.25 μg/mL of ZNF703 antibody (Abcam ab40861) followed by 0.06 μg/mL of GAPDH antibody (RDI) that was used as a loading control prior to ECL development and exposure to film. The antibody detected the full-length 58.2 kDa isoform that has been approved by the CCDS database (http://www.ncbi.nlm.nih.gov/CCDS/CcdsBrowse.cgi). The films were scanned and the protein was quantified in Photoshop CS2 (Adobe). The absolute intensity of a protein band was obtained by subtracting the background from the value of the protein band and multiplying by the pixel value. The absolute value of each protein band was divided by the respective value of GAPDH, which allowed comparison of the relative protein expression within each gel. Cell lysate from the breast cancer cell line SUM52, which was cultured under the condition recommended by Asterand, was included on all gels to allow comparison between gels.

### Statistical analysis

Statistical analyses, including the survival analyses using the Survival package, were performed in R 31 (http://www.r-project.org) version 2.11.1. Correlation of ZNF703 mRNA level with clinical, molecular, or tumor characteristics was performed for 577 samples unless otherwise specified. Three-hundred and fifty-nine tumors, a subset of the 577 samples, were used for correlation analysis of ZNF703 mRNA levels with *ZNF703* copy number due to the limiting number of tumors with array-CGH data. The ZNF703 mRNA levels were analyzed according to *ZNF703* copy number status using nonparametric analysis. The Pearson correlation coefficient was calculated to analyze correlation between protein quantity and mRNA expression. Correlation of ZNF703 mRNA levels with molecular subtypes, histopathology, and clinical parameters was performed with parametric tests, Student's *t*-test, or analysis of variance (ANOVA).

To examine the effect of ZNF703 mRNA expression on survival in patients carrying ER-positive luminal (combined luminal A and B) or luminal B tumors, the tumors were divided into high expressing tumors, whose relative expression of ZNF703 mRNA was ≥ the mean and tumors expressing low ZNF703 mRNA, that is, below the mean. The survival curves were compared using Kaplan–Meier estimates and the log rank test.

## Results

### *ZNF703* is the only gene within the smallest region of overlap at 8p12

**Figure 1:**
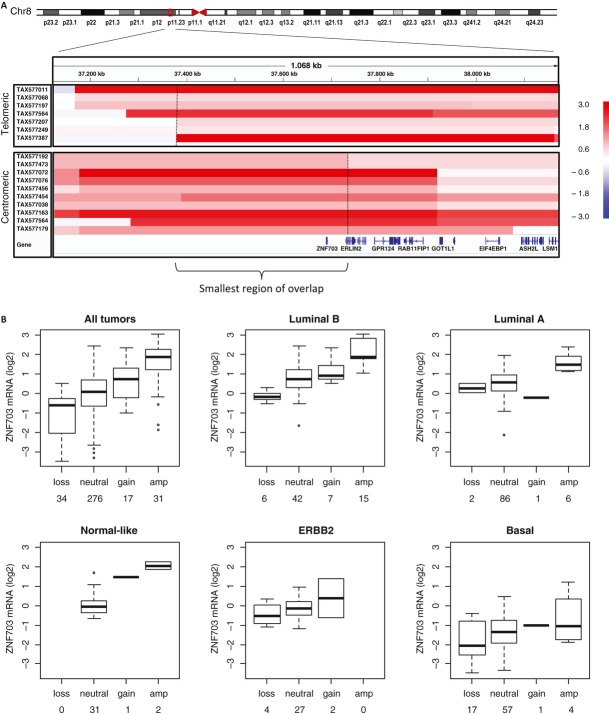
*ZNF703*, the only full-length gene within the smallest region of overlap (SRO), exhibits the best correlation between DNA and mRNA levels in tumors of the luminal B subtype. (A) The SRO at the amplified 8p12 region included *ZNF703*. The CNAs between 37.1 and 38.2 kb from selected tumors are shown. The IGV heatmap (http://www.broadinstitute.org/igv 36–37) shows the alignment of the copy number data from the indicated tumors, which was used to define the telomeric and centromeric border of the SRO. The telomeric and centromeric boundaries of the SRO within 8p12-p11 mapped to genomic positions 37368027 and 37717593, respectively (hg 17). The black dotted lines and bracket demarcate the SRO. The scale on the right depicts the log_2_ transformed values for the BACs. (B) Tumors were placed into the categories loss, neutral, gain, and amplification (amp) depending on the copy number values. The number of tumors in each category is indicated below the plot. A Kruskal–Wallis analysis was performed to analyze the correlation between mRNA expression and DNA copy number for the whole group and in the individual subtypes: all tumors, *P* = 8.7 × 10^−16^; luminal B subtype, *P* = 1.2 × 10^−7^; luminal A, *P* = 1.7 × 10^−3^; normal like, *P* = 0.02; ERBB2,* P* = 0.6; and basal, *P* = 0.3.

CNAs were mapped by array-CGH hybridizations of the genomes of 359 breast tumors from Nordic patients. There was amplification (log_2_ ratio >0.85) within the 8p12-p11 locus in 41 (11.4%) tumors. Amplification of the telomeric 8p12 region was most frequently observed, 31 (75%) of the 41 tumors. These 31 tumors were used to define the SRO that was amplified. The telomeric boundary of the SRO was upstream of *ZNF703* (Fig. 1A) in a region where no known genes reside (hg17 32). Two luminal B tumors, TAX577473 and TAX577192, define the centromeric boundary that was demarcated by a break between amplification (log_2_ ratio 1) and gain (log_2_ ratio 0.6). The boundary falls within intron 3 of the *ERLIN2* gene, leaving inside the SRO, *ZNF703*, and a noncoding exon 1 as well as exons 2 and 3 of the *ERLIN2* gene that code for 69 amino acids. The SRO thus encompassed a genetic region whose only full-length gene was *ZNF703*. In tumor samples TAX577473 and TAX577192 there was high expression from *ZNF703* but not from *ERLIN2*. Hence, *ERLIN2* was excluded as the genetic driver of the telomeric 8p12 amplicon.

### ZNF703 protein and mRNA expression correlate in tumors of the luminal B subtype

The 359 Nordic tumors used for analysis of correlation between ZNF703 mRNA and copy number represented the molecular subtypes – 79 basal, 34 ERBB2, 95 luminal A, 70 luminal B, 34 normal like, and 47 unclassified. Amplification of *ZNF703* was observed in 4 (5.1%) of 79 basal tumors, none of 34 (0%) ERBB2 tumors, 6 (6.3%) of 95 luminal A tumors, 15 (21.4%) of 70 luminal B tumors, 2 (5.9%) of 34 normal-like tumors, and 4 (8.5%) of 47 unclassified tumors. Thus, the dominant subtype with amplification of *ZNF703* was the luminal B subtype (48% of tumors), with the remaining amplified tumors of subtypes luminal A (19%), basal (13%), normal like (7%), and unclassified (13%) (Fig. S1). The ZNF703 mRNA levels were highest in luminal B tumors (Fig. S2). Categorization of tumors according to mRNA expression into high expression (>1 SD, *n* = 39) and normal expression (≤1 SD, *n* = 320) revealed that 0% of basal (0/79), 0% of ERBB2 (0/34), 9.5% of luminal A (9/95), 31.4% of luminal B (22/70), 11.8% of normal like (4/34), and 8.5% of unclassified (4/47) tumors expressed high ZNF703 mRNA levels. Therefore, 56.4% or 22 of the 39 tumors that expressed high levels of ZNF703 mRNA were of the luminal B subtype. Correlation of ZNF703 mRNA with *ZNF703* copy number categorized according to amplification, gain, loss, or neutral status revealed the strongest correlation in the luminal B (*P* = 1.2 × 10^−7^) subtype followed by the luminal A (1.7 × 10^−3^) and normal-like (*P* = 0.02) subtypes, but no correlation was observed in the ERBB2 or basal subtypes (Fig. 1B). From these analyses it became clear that some of the tumors that were copy neutral for *ZNF703* also expressed high levels of ZNF703 mRNA.

**Figure 2:**
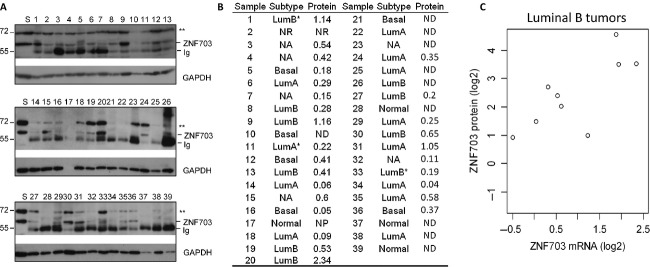
Protein expression from *ZNF703* correlated positively with mRNA expression in luminal B tumors. (A) ZNF703 protein expression was analyzed by Western blotting in 39 breast tumors. The Western blots were probed with antiserum against ZNF703 and GAPDH as a loading control. S stands for the breast cancer cell line SUM52 and the tumor samples are numbered from 1 to 39. Molecular weight (kDa) markers are shown on the left. The molecular weight of the full-length ZNF703 protein is 58.2 kDa. **Nonspecific binding. Ig denotes the detection of immunoglobulins by the secondary goat antiserum against the primary ZNF703 antiserum. (B) The table shows the sample identifiers, subtype, and the relative protein level of ZNF703. The tumors labeled with * most closely matched the indicated subtype (see Materials and Methods). The quantification of ZNF703 with respect to GAPDH was as described in methods. ND denotes that no ZNF703 expression was detected, NP means that no protein was observed in the lane, and NR is not relevant (not breast tumor). No protein expression (ND) was not due to no or little mRNA expression in the tumor. (C) Correlation analysis between log_2_ transformed ZNF703 protein and mRNA levels in luminal B tumors. The Pearson correlation coefficient was *r* = 0.75, *P* = 0.02.

To determine whether the correlation between *ZNF703* copy number and mRNA level reflected protein level, protein expression was analyzed by Western blotting in 39 tumors, including 12 luminal A tumors, 10 luminal B tumors, and 4 normal-like tumors (Fig. 2). Only the large isoform (58.2 kDa) of ZNF703 was observed. A significant positive correlation between ZNF703 mRNA and protein levels was observed in luminal B tumors (*r* = 0.75, *P* = 0.02; Fig. 2C) but not in tumors of the luminal A subtype (data not shown). Expression of ZNF703 protein was significantly higher in luminal B tumors than in luminal A tumors (*P* = 0.008). ZNF703 protein expression was not detected in the normal-like tumors. Although the number of tumors is small the results suggest a correlation between DNA amplification and gene expression of *ZNF703* at the protein level in luminal B tumors.

### High ZNF703 mRNA levels correlate with shorter survival

The correlation of ZNF703 mRNA with clinical and patho-logical characteristics in 577 Nordic tumors 29 confirmed highest expression in tumors of the luminal B subtype and the lowest in the basal subtype (*P* = 2 × 10^−16^; Fig. S2). Also, ZNF703 mRNA expression was higher in hormone receptor–positive tumors (*P* = 2 × 10^−16^). Luminal A and luminal B tumors constitute the majority of the ER-positive tumors. They were also the subtypes in which DNA copy number and mRNA levels were most significantly correlated. We therefore analyzed the effect of ZNF703 mRNA expression on prognosis in these subtypes by testing for a potential correlation of ZNF703 mRNA expression with clinical and pathological parameters.

**Figure 3:**
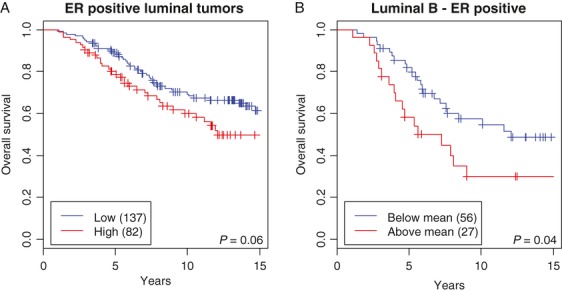
High ZNF703 mRNA is correlated with shorter survival in patients with luminal B tumors. The overall survival was examined in patients with (A) ER-positive luminal A and B tumors combined and (B) in ER-positive luminal B tumors separately. The patients were divided into two groups according to tumor ZNF703 mRNA levels: low expressing tumors (<below mean) and high expressing tumors (≥mean) based on the mean ZNF703 mRNA value in all luminal or luminal B tumors. The number of patients in each expression group is shown along with the log rank *P*-values. For patients with luminal B tumors, the median survival time was 12.1 and 6.4 years with low and high ZNF703 mRNA, respectively.

**Figure 4:**
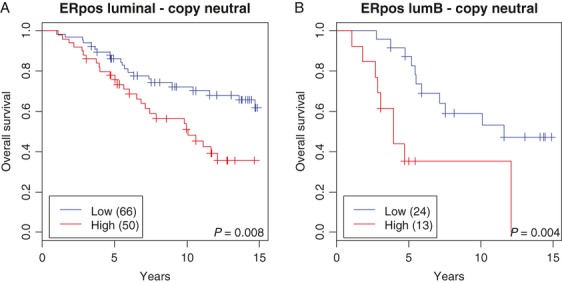
High ZNF703 mRNA level is correlated with shorter overall survival (OS) in patients with *ZNF703* copy number neutral, ER-positive, luminal B tumors. OS was analyzed in patients with ER-positive (A) luminal or (B) luminal B *ZNF703* copy neutral tumors. Patients were divided into groups according to tumor ZNF703 mRNA: low expression (<mean) and high expression (≥mean). The number in each group and log rank *P*-values are indicated in the figures. The median survival time was 11.6 and 4.0 years for patients with ER-positive luminal B tumors expressing low or high ZNF703 mRNA, respectively.

Correlation between histoclinical parameters and ZNF703 mRNA levels in ER-positive luminal tumors revealed higher ZNF703 mRNA levels in PR-negative tumors (*P* = 0.008), a gradual increase in mRNA according to histological grade (*P* = 0.04), and an increased number of deaths was observed in patients with tumors containing high levels of ZNF703 mRNA (*P* = 0.002, Table 1). No difference was observed in ZNF703 mRNA according to the histological origin of the tumor or age at diagnosis. The significant difference in ZNF703 mRNA according to BRCA mutation status was due to higher expression in *BRCA2*-mutated tumors as compared with sporadic tumors, which is explained by the high ratio of *BRCA2*-mutated ER-positive luminal tumors belonging to the luminal B subtype (80%). There was a trend toward shorter overall survival (OS) in patients with ER-positive luminal tumors, expressing high (above the mean) ZNF703 mRNA levels (log rank *P* = 0.06, Fig. 3A). Half of the ER-positive luminal B tumors expressed high levels of ZNF703 mRNA, but 31% of the ER-positive luminal A tumors. Survival analysis of the luminal subtypes separately showed that high ZNF703 mRNA led to shorter OS in luminal B tumors (log rank *P* = 0.04, Fig. 3B), whereas a significant difference was not observed in luminal A tumors (data not shown). The hazard ratio (HR) for ZNF703 mRNA in luminal B tumors was 1.90 (95% confidence interval [CI] 1.02, 3.54, *P* = 0.04), but an adjustment for the histoclinical parameters could not be performed due to low number of events 33. A correlation between ZNF703 expression levels and tumor characteristics was not observed in the luminal B or luminal A subtypes (Table S2). Some of the *ZNF703* copy neutral luminal A and B tumors express very high levels of ZNF703 mRNA (Fig. 1B), which prompted the separate analysis of these tumors. A significantly shorter OS was observed in ER-positive luminal tumors (*P* = 0.008, Fig. 4A) which was attributed to ER-positive luminal B tumors (*P* = 0.004, Fig. 4B). High expression of ZNF703 was significantly associated with shorter survival in ER-positive luminal tumors (HR 2.28, CI 1.30, 3.99, *P* = 0.004), and after adjustment for PR expression (HR 2.17, CI 1.19, 3.93, *P* = 0.01; Table S3).

**Table:** Correlation of ZNF703 mRNA levels in ER-positive luminal A and luminal B tumors with clinical and pathological parameters

ER pos luminal tumors	*n* = 221	ZNF703 mRNA level, mean (±SD)	*P*-value
Histopathology			
IDC	150	1.98 (1.36)	0.26
ILC	16	1.88 (1.01)	
Other	29	1.73 (1.14)	
Unknown	26		
Histologic grading			
1	27	1.49 (0.71)	0.04
2	76	1.89 (1.35)	
3	37	2.32 (1.64)	
Unknown	81		
Progesterone receptor			
Negative	39	2.70 (1.94)	0.008
Positive	178	1.77 (1.11)	
Unknown	4		
Age			
<50	97	1.90 (1.17)	0.50
≥50	119	1.97 (1.47)	
Unknown	5		
Mutation status			
BRCA 1	2	2.64 (0.78)	0.0003
BRCA 2	25	2.55 (1.25)	
BRCA X	78	2.10 (1.52)	
Other	8	2.78 (2.43)	
Sporadic	108	1.60 (0.99)	
Death			
No	136	1.75 (1.15)	0.002
Yes	84	2.26 (1.53)	
Unknown	1		

IDC, invasive ductal tumors; ILC, invasive lobular tumors. The mean values of ZNF703 mRNA level are shown along with SD. The *P*-values were calculated with a *T*-test (two variables) or an ANOVA (>2 variables) using the log_2_ transformed ZNF703 mRNA levels.

## Discussion

Our results support previous studies that demonstrated *ZNF703* as an oncogene with a role in luminal B tumors 16–17. Furthermore, we show that high ZNF703 mRNA resulted in shorter OS in patients with ER-positive luminal B tumors, regardless of amplification. These results suggest worse prognosis in patients with luminal B tumors that express high level of *ZNF703*.

*ZNF703* has been indicated as the genetic driver of the amplification at 8p12 in luminal B tumors 16–17. In one study the gene was identified when comparing genomic alterations and gene expression between luminal A and B tumors 17. In another study, *ZNF703* was the only gene within the SRO 16. Similarly in our study, *ZNF703* was the only full-length gene within the SRO in two luminal B tumors that had a centromeric break point within *ERLIN2*. The previous studies 16–17 provided support for *ZNF703* being a class II oncogene in luminal B breast tumors in keeping with a scheme that classifies amplified cancer genes according to importance in cancer development 34. In accord with this scheme, our results support *ZNF703* as a class III oncogene 34. Consistent with the classical definition of an oncogene there was correlation between gene amplification, mRNA, and protein expression of *ZNF703* in luminal B tumors. A positive correlation between amplification and mRNA expression of *ZNF703* in breast tumors has not been observed consistently. This is most likely due to heterogeneity of breast tumors, tumor sample sets, and the fact that the strongest correlation is in ER-positive 16 or in luminal B tumors 17, as observed in this study, as well as lower or nonexistent correlation in other subtypes such as the ERBB2 and basal subtypes. Correlation with protein expression, as measured by immunohistochemistry, has been reported in one study in which protein expression was higher in ER-positive tumors with amplification 16. We measured protein expression by Western blotting and observed the full-length isoform of ZNF703. A positive correlation between protein and mRNA levels was observed in luminal B tumors, which supports *ZNF703* as an oncogene in these tumors. However, the number of luminal B tumors available to us was small and a higher number of luminal B tumors are needed for confirmation. In a recent study that identified genetic drivers based on the genomic and transcriptomic landscape in breast tumors, *ZNF703* was identified as a genetic driver in some tumors of the luminal B, luminal A, and normal-like subtypes 18. From our data we can neither confirm nor preclude *ZNF703* as a genetic driver in normal-like and luminal A tumors. Protein expression in luminal A tumors was lower than in luminal B tumors and thus the correlation between mRNA and protein may be weak and a larger sample size would be necessary to observe a positive correlation. Protein expression was not detected in the normal-like tumors preventing correlation analysis with mRNA.

High ZNF703 mRNA expression has been shown to associate with shorter disease-free survival in a combined analysis of luminal A and luminal B tumors 17. We confirm this observation by showing a trend in shorter OS in patients with ER-positive luminal tumors expressing high ZNF703 mRNA. Additionally, we demonstrate that high ZNF703 mRNA was associated with shorter OS in patients with luminal B tumors (Fig. 3B), an observation not previously reported. The mean age at diagnosis was 51 years (median age 48 years, range 26–88 years) and thus the OS analyses were restricted to 15 years to decrease the likelihood of deaths due to other causes. Information regarding breast cancer–specific survival and distant metastasis-free survival was not available for the whole group of patients.

Patients with *ZNF703* copy neutral, ER–positive, luminal tumors expressing high ZNF703 mRNA had worse prognosis than those with tumors expressing low ZNF703 mRNA. This finding was due to patients with ER-positive luminal B tumors (Fig 4B). To our knowledge, this has not been reported before but it appears that ZNF703 expression above a certain threshold can impart a malignant character to a cell. Thus, CNA may not be the only mechanism by which the expression of ZNF703 is deregulated. At a cellular level, high expression from the *ZNF703* gene has been shown to activate the E2F1 transcription profile and downregulate the ER transcription profile which was proposed to push the tumors toward a stem-like path (proliferation) rather than a differentiation path 17. An increase in *ZNF703* expression may also induce transcription of growth factors or components of growth factor pathways that enhance the proliferative or metastatic potential of cells. Activation of growth factors in luminal B breast cancer has been suggested as a reason for resistance to therapy and early relapse (reviewed in Tran and Bedard 35).

Taken together, our results support *ZNF703* as an oncogene in breast cancer whose high expression predisposes to poor clinical outcome in patients with luminal B tumors. We suggest *ZNF703* as a candidate for a marker of worse prognosis, in particular for women with luminal B breast cancer.

## Supporting Information

**Figure S1.** Amplification of *ZNF703* was most often observed in tumors of the luminal B subtype. Amplification of *ZNF703* was observed in 31 (8.6%) of the 359 breast tumors with available information on molecular subtypes. Of these 13% (4/31) were of the basal subtype, 0% (0/31) ERBB2, 19% (6/31) luminal A, 48% (15/31) luminal B, 7% (2/31) normal like, and 13% (4/31) unclassified.

**Figure S2. **ZNF703 mRNA expression was observed in tumors of the luminal B subtype. Comparison of ZNF703 mRNA expression between the molecular subtypes of breast cancer (basal [123], ERBB2 [59], luminal A [149], luminal B [95], normal like [78], and unclassified [73]) showed that the expression is highest in the luminal subtypes and lowest in the basal subtype. The highest expression occurred in the luminal B tumors, *P* = 2 × 10^−16^.

**Table S3.** Hazard ratio (HR) and 95% confidence interval (CI) for *ZNF703* expression in copy neutral ER-positive luminal tumors.
