# Supplementary figures and images for: High expression of ZNF703 independent of amplification indicates worse prognosis in patients with luminal B breast cancer
Source: Cancer Med. 2013 May 22;2(4):437–46. doi: 10.1002/cam4.88 (PMC3799278; doi:10.1002/cam4.88)

■ Basal ■ ERBB2 ■ Luminal A ■ Luminal B ■ Normal-like ■ Unclassified

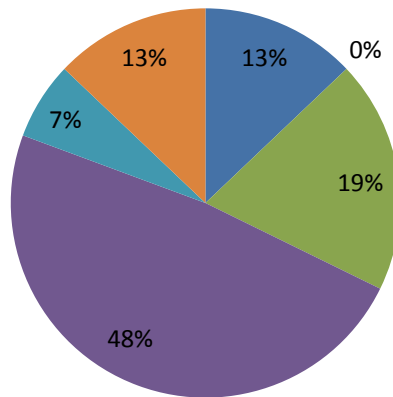

Supplement: Supplementary file 1 — Figure S1. Amplification of ZNF703 was most often observed in tumors of the luminal B subtype. Amplification of ZNF703 was observed in 31 (8.6%) of the 359 breast tumors with available information on molecular subtypes. Of these 13% (4/31) were of the basal subtype, 0% (0/31) ERBB2, 19% (6/31) luminal A, 48% (15/31) luminal B, 7% (2/31) normal like, and 13% (4/31) unclassified. [file cam40002-0437-SD1.pdf]

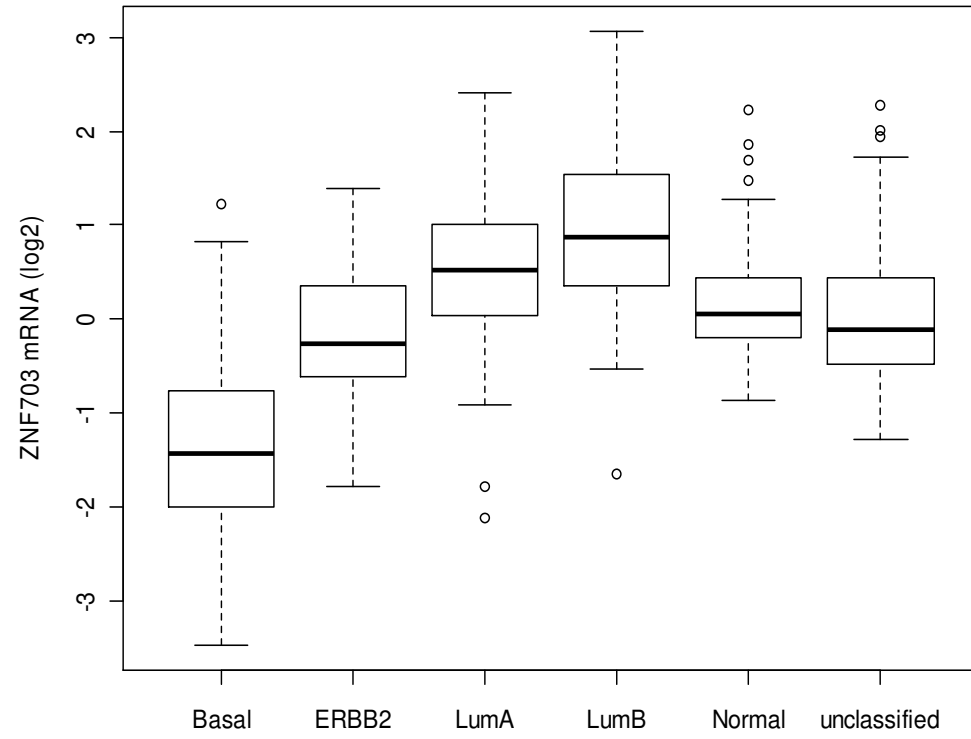

Supplement: Supplementary file 2 — Figure S2. ZNF703 mRNA expression was observed in tumors of the luminal B subtype. Comparison of ZNF703 mRNA expression between the molecular subtypes of breast cancer (basal [123], ERBB2 [59], luminal A [149], luminal B [95], normal like [78], and unclassified [73]) showed that the expression is highest in the luminal subtypes and lowest in the basal subtype. The highest expression occurred in the luminal B tumors, P = 2 × 10^−16^. [file cam40002-0437-SD2.pdf]
