# Supplementary material for: High expression of ZNF703 independent of amplification indicates worse prognosis in patients with luminal B breast cancer
Source: Cancer Med. 2013 May 22;2(4):437–46. doi: 10.1002/cam4.88 (PMC3799278; doi:10.1002/cam4.88)
Supplement: Supplementary file 3 [file cam40002-0437-SD3.doc]

**Supplementary Table 1** Clinical, pathology and molecular characteristics for the 577 tumors.

|  |  | **n (%)** |
| --- | --- | --- |
| **Histopathology** |  |  |
|  | IDC | 373 (65) |
|  | ILC | 41 (7) |
|  | other | 66 (11) |
|  | unknown | 97 (17) |
| **Histologic grading** | |  |
|  | 1 | 48 (8) |
|  | 2 | 155 (27) |
|  | 3 | 187 (32) |
|  | unknown | 187 (32) |
| **Estrogen receptor** | |  |
|  | negative | 194 (34) |
|  | positive | 338 59) |
|  | unknown | 45 (8) |
| **Progesterone receptor** | |  |
|  | negative | 231 (40) |
|  | positive | 297 (51) |
|  | unknown | 49 (8) |
| **Age** |  |  |
|  | Median age, years (range) | 48 (26 - 88) |
|  | < 50 | 298 (52) |
|  | ≥ 50 | 249 (43) |
|  | unknown | 30 (5) |
| **Mutation status** | |  |
|  | BRCA1 | 34 (6) |
|  | BRCA 2 | 39 (7) |
|  | BRCA X | 195 (34) |
|  | Other | 36 (6) |
|  | Sporadic | 293 (51) |
| **Subtype** |  |  |
|  | Basal | 123 (21) |
|  | ERBB2 | 59 (10) |
|  | Luminal A | 149 (26) |
|  | Luminal B | 95 (16) |
|  | Normal | 78 (14) |
|  | unclassified | 73 (13) |
| **Overall survival (OS)** | |  |
|  | Median OS in years (range) | 8.2 (0.12 - 24.5) |
