# Supplementary material for: High expression of ZNF703 independent of amplification indicates worse prognosis in patients with luminal B breast cancer
Source: Cancer Med. 2013 May 22;2(4):437–46. doi: 10.1002/cam4.88 (PMC3799278; doi:10.1002/cam4.88)
Supplement: Supplementary file 4 [file cam40002-0437-SD4.docx]

**Supplementary Table 2** Correlation of ZNF703 mRNA levels with clinical-pathological parameters in ER positive luminal B and luminal A tumors. The mean values for *ZNF703* mRNA level are shown along with the standard deviation (SD). The p-values were calculated with t-test (2 variables) or ANOVA (more than 2 variables) using the log_2_ transformed *ZNF703* mRNA levels.

|  | **ER pos LumB tumors** | **n** | **ZNF703 mRNA** | **p-value** |  |  | **ER pos LumA tumors** | **n** | **ZNF703 mRNA** | **p-value** |
| --- | --- | --- | --- | --- | --- | --- | --- | --- | --- | --- |
|  |  | 84 | mean (±SD) |  |  |  |  | 137 | mean (±SD) |  |
| **Histopathology** | |  |  |  |  | **Histopathology** | |  |  |  |
|  | IDC | 62 | 2.45 (1.76) | 0.22 |  |  | IDC | 88 | 1.64 (0.85) | 0.60 |
|  | ILC | 2 | 3.43 (2.47) |  |  |  | ILC | 14 | 1.65 (0.54) |  |
|  | other | 12 | 1.96 (1.46) |  |  |  | other | 17 | 1.56 (0.85) |  |
|  | unknown | 8 |  |  |  |  | unknown | 18 |  |  |
| **Histologic grading** | | |  |  |  | **Histologic grading** | | |  |  |
|  | 1 | 4 | 1.50 (0.60) | 0.47 |  |  | 1 | 23 | 1.49 (0.75) | 0.72 |
|  | 2 | 23 | 2.55 (2.07) |  |  |  | 2 | 52 | 1.60 (0.73) |  |
|  | 3 | 25 | 2.60 (1.84) |  |  |  | 3 | 12 | 1.74 (0.93) |  |
|  | unknown | 32 |  |  |  |  | unknown | 50 |  |  |
| **Progesterone receptor** | | |  |  |  | **Progesterone receptor** | | |  |  |
|  | negative | 23 | 3.02 (2.27) | 0.22 |  |  | negative | 16 | 2.24 (1.25) | 0.06 |
|  | positive | 61 | 2.22 (1.54) |  |  |  | positive | 117 | 1.53 (0.69) |  |
|  | unknown | 0 |  |  |  |  | unknown | 4 |  |  |
| **Age** |  |  |  |  |  | **Age** |  |  |  |  |
|  | < 50 | 41 | 2.26 (1.55) | 0.73 |  |  | < 50 | 56 | 1.64 (0.68) | 0.33 |
|  | ≥ 50 | 42 | 2.64 (2.01) |  |  |  | ≥ 50 | 77 | 1.60 (0.90) |  |
|  | unknown | 1 |  |  |  |  | unknown | 4 |  |  |
| **Mutation status** | |  |  |  |  | **Mutation status*** | | |  |  |
|  | BRCA1 | 1 | 3.19 (NA) | 0.37 |  |  | BRCA1 | 1 | 2.09 (NA) | 0.02 |
|  | BRCA 2 | 20 | 2.67 (1.29) |  |  |  | BRCA 2 | 5 | 2.09 (1.08) |  |
|  | BRCA X | 28 | 2.58 (2.11) |  |  |  | BRCA X | 50 | 1.83 (0.97) |  |
|  | Other | 3 | 3.63 (4.14) |  |  |  | Other | 5 | 2.29 (0.96) |  |
|  | Sporadic | 32 | 2.04 (1.48) |  |  |  | Sporadic | 76 | 1.42 (0.61) |  |
| **Death** |  |  |  |  |  | **Death** |  |  |  |  |
|  | No | 41 | 2.13 (1.67) | 0.07 |  |  | No | 95 | 1.59 (0.80) | 0.13 |
|  | Yes | 43 | 2.73 (1.87) |  |  |  | Yes | 41 | 1.76 (0.85) |  |
|  | unknown | 0 |  |  |  |  | unknown | 1 |  |  |
