# Supplementary material for: High expression of ZNF703 independent of amplification indicates worse prognosis in patients with luminal B breast cancer
Source: Cancer Med. 2013 May 22;2(4):437–46. doi: 10.1002/cam4.88 (PMC3799278; doi:10.1002/cam4.88)
Supplement: Supplementary file 5 — Table S3. Hazard ratio (HR) and 95% confidence interval (CI) for ZNF703 expression in copy neutral ER-positive luminal tumors. [file cam40002-0437-SD5.docx]

**Supplementary Table 3.** Hazard ratio (HR) and 95% confidence interval (CI) for ZNF703 expression in copy neutral ER positive luminal tumors.

|  | **Univariate analysis** | | |
| --- | --- | --- | --- |
|  | HR | 95% CI | p-value |
| ZNF703 mRNA | 2.28 | 1.30 - 3.99 | 0.004 |
| Age | 1.47 | 0.83 - 2.61 | 0.19 |
| PR | 0.4 | 0.20 - 0.78 | 0.007 |
| Grade |  |  |  |
| 2 | 0.91 | 0.34 - 2.47 | 0.86 |
| 3 | 1.73 | 0.55 - 5.50 | 0.35 |
|  |  |  |  |
|  | **Multivariate analysis** | | |
|  | HR | 95% CI | p-value |
| ZNF703 mRNA | 2.17 | 1.19 - 3.93 | 0.01 |
| PR | 0.54 | 0.27 - 1.11 | 0.09 |

The number of events per variable were too few to include all of the histoclinical parameters in the multivariate analysis (Peduzzi P., et al., J Clin Epidemiol Vol. 48, No. 12, pp. 1503 – 1510, 1995). Thus, an adjustment was made only for the expression of the progesterone receptor (PR) due to its significant effect in the univariate analysis.
